# Causal effects and metabolites mediators between immune cell and risk of colorectal cancer: a Mendelian randomization study

**DOI:** 10.3389/fimmu.2024.1444222

**Published:** 2024-09-12

**Authors:** Qian Yang, Bixia Duan, Jian Yue, Donglin Zhang, Xueping Chen, Mengjia Shi, Jie Kan, Ruochan Li, Hongda Li, Lin Gan

**Affiliations:** ^1^ Clinical Molecular Medicine Testing Center, The First Affiliated Hospital of Chongqing Medical University, Chongqing, China; ^2^ Chongqing Key Laboratory of Molecular Oncology and Epigenetics, The First Affiliated Hospital of Chongqing Medical University, Chongqing, China; ^3^ Institute for Brain Science and Disease, Key Laboratory of Major Brain Disease and Aging Research (Ministry of Education), Chongqing Medical University, Chongqing, China; ^4^ Department of Oncology, The Affiliated Yongchuan Hospital of Chongqing Medical University, Chongqing, China; ^5^ Department of Breast Surgery, Gaozhou People’s Hospital, Gaozhou, China; ^6^ Department of General, Visceral, and Transplant Surgery, Ludwig-Maximilians-University Munich, Munich, Germany; ^7^ Biobank Center, The First Affiliated Hospital of Chongqing Medical University, Chongqing, China

**Keywords:** immune cell, metabolites, Mendelian randomization, colorectal cancer, mediation analysis

## Abstract

**Objective:**

The involvement of immune cells in colorectal cancer (CRC) and their interplay with metabolic disorders are yet to be fully elucidated. This study examines how peripheral immune cells, inferred genetically, affect CRC and investigates the intermediary roles of metabolites.

**Methods:**

We employed a two-sample bidirectional Mendelian randomization (MR) approach to assess the causal influence of immune cells on CRC. Additionally, a two-step MR strategy was utilized to pinpoint potential metabolites that mediate this effect. Our analysis incorporated data from genome-wide association studies (GWAS), involving 731 immune cell types, 1,400 metabolites, and CRC outcomes. The primary method of analysis was randomized inverse variance weighting (IVW), supported by MR-Egger, weighted median, simple mode, and weighted mode analyses. Sensitivity checks were conducted using Cochran’s Q test, MR-PRESSO test, MR-Egger regression intercept, and leave-one-out analysis.

**Results:**

The study identified 23 immune cell types and 17 metabolites that are causally linked to CRC. Our mediation analysis highlighted that nine metabolites act as intermediaries in the relationship between nine specific immune cells and CRC risk. Notably, The ratios of Adenosine 5’-monophosphate (AMP) to aspartate and Retinol (Vitamin A) to linoleoyl-arachidonoyl-glycerol (18:2 to 20:4) were found to concurrently mediate the promoting effects of Myeloid DC %DC and BAFF-R on B cells in colorectal cancer (CRC). Moreover, iminodiacetate (IDA) was found to mediate the protective effect of CD14^+^ CD16^-^ monocytes on CRC, contributing 11.8% to this mediation. In contrast, IDA was also seen to decrease the protective effect of IgD^+^ CD38br %B cells on CRC risk, with a mediation effect proportion of -10.4%.

**Conclusion:**

This study delineates a complex network involving immune cells, metabolites, and CRC, suggesting a multifaceted pathophysiological interaction. The identified causal links and mediation pathways underscore potential therapeutic targets, providing a foundation for interventions aimed at modulating immune responses to manage CRC.

## 1 Introduction

CRC is one of the most common malignancies of the digestive system, consistently showing high incidence rates in recent years. In 2022, there were over 1.92 million new cases and more than 900,000 deaths worldwide, with a 5-year survival rate of 65% (1, 2). The etiology of CRC is multifaceted, involving both genetic predispositions—such as Lynch syndrome (3) and inflammatory bowel disease (4)—and environmental influences, including lifestyle factors like smoking (5), excessive alcohol consumption (6), obesity (7), and gut microbiota composition (8). Immune cells are integral to the pathogenesis of CRC. Research has specifically implicated macrophages (9), dendritic cells (10), T cells (11), and NK cells (12) in the initiation and progression of CRC, with their precise mechanisms still needing clarification.

Multiple studies have reported that metabolic reprogramming within immune cells plays a critical role in tumor malignancy, influencing the effectiveness of anti-tumor responses (13). For example, inhibiting glycolysis in CD8^+^ T cells not only enhances their memory function but also boosts their anti-tumor activity (14). Similarly, alterations in amino acid metabolism, particularly involving arginine and glutamine, significantly affect T cell viability and functionality, thereby modulating their capacity to combat tumors (15, 16). Curtailing cholesterol metabolism in CD8^+^ T cells has also been shown to enhance their anti-tumor capabilities (17). However, a reduction in glycolysis in NK cells has been linked to accelerated lung cancer progression (18). These findings underscore the central role of immune cell metabolism in anti-tumor immunity. Moreover, additional studies suggest that targeting specific metabolic pathways, such as fatty acid metabolism in myeloid-derived suppressor cells (19), ketone metabolism in macrophages (20), and cholesterol efflux in macrophages (21), could profoundly influence tumor dynamics. These studies provide evidence from animal experiments and mechanistic insights into how metabolic reprogramming of immune cells influences tumor progression; however, evidence from large-scale human studies remains limited.

Mendelian randomization uses genetic variants as instrumental variables to assess causal relationships between exposures and outcomes, effectively simulating the conditions of a controlled experiment. This approach offers distinct advantages over both observational studies and randomized controlled trials (RCTs). Unlike observational studies, which are often plagued by confounding and reverse causation, Mendelian randomization leverages naturally occurring genetic variations that are randomly assigned and independent of the outcome, significantly reducing these biases. Compared to RCTs, which may be impractical due to ethical or economic limitations, Mendelian randomization provides a viable and efficient alternative for exploring causal relationships without the need for direct intervention (22).

Although recent Mendelian analyses have shown that metabolite-related SNPs can modulate the role of immune cells in the development of various cancers, including breast, pancreatic, and hepatocellular carcinoma (23–25), these studies have not fully addressed the complexities of immune cell involvement in colorectal cancer. Moreover, prior research about immune cells and colorectal cancer has primarily focused on mechanistic insights, lacking the perspective of large-scale genetic variation, which limits their ability to draw robust causal conclusions. Furthermore, while one Mendelian randomization study has explored the relationship between peripheral blood cell counts and colorectal cancer risk, identifying eosinophils and lymphocytes as potential contributors, this study examined only five leukocyte counts without delving into the specific roles of immune cell subtypes (26). Consequently, there still remains a gap in understanding the contribution of immune cells to colorectal cancer risk, particularly regarding the mediating role of metabolites.

In contrast, our study addresses these limitations by investigating the contribution of immune cells to colorectal cancer risk through a novel approach that integrates large-scale genetic variation and examines the mediating role of metabolites. This approach not only broadens the understanding of immune cell involvement in colorectal cancer but also provides new insights into the complex interplay between metabolites and immune cells, thereby filling a critical gap in the existing literature.

## 2 Methods

### 2.1 Study design

**Figure 1:**
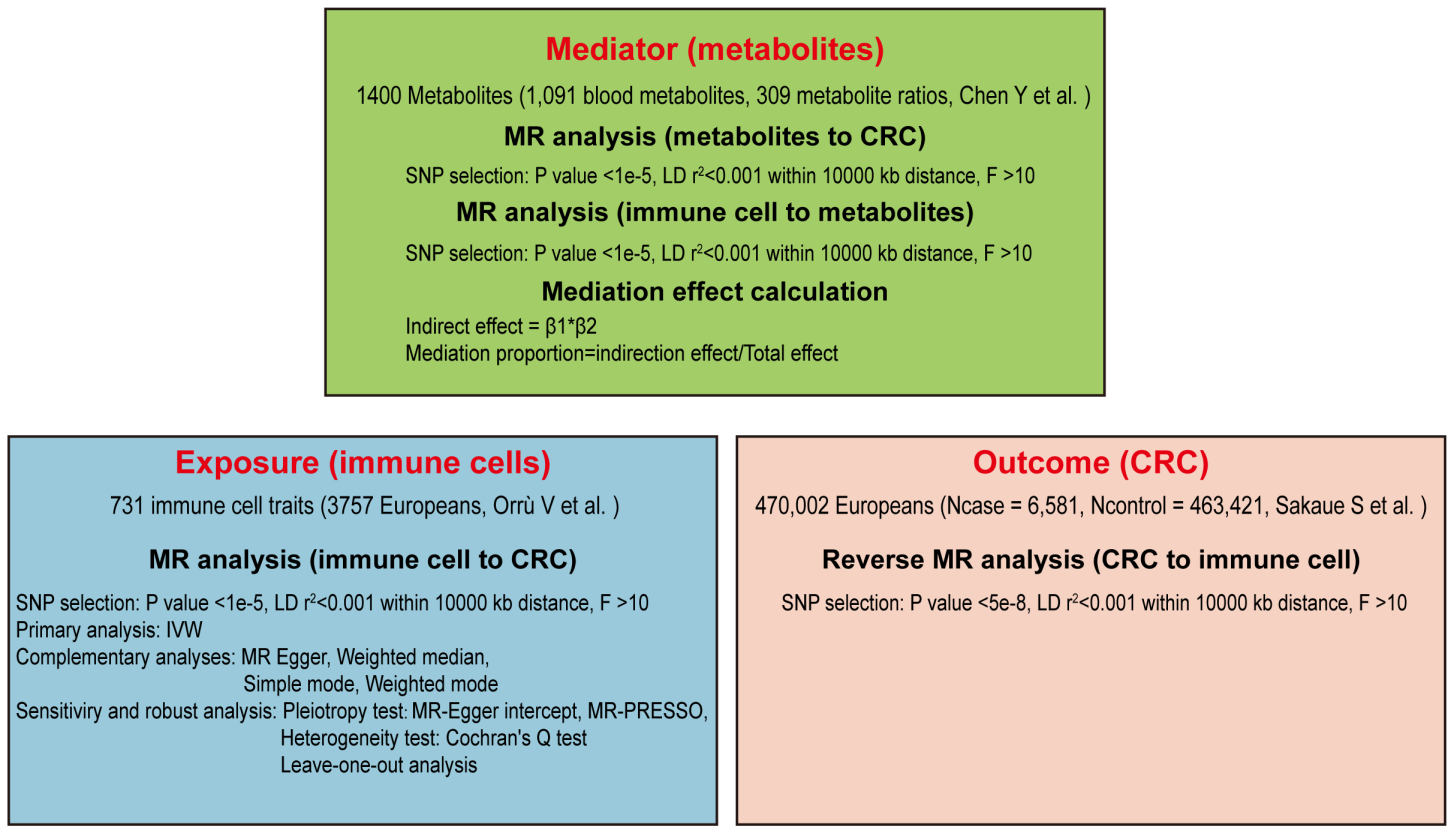
Flow chart of the Mendelian randomization analysis.

Our study aims to clarify the causal connections between immune cells and the risk of CRC through a detailed Mendelian randomization analysis. Initially, we screened 731 immune cell traits for causal associations with CRC using a two-sample MR approach. Specific immune cells were then selected for further analysis, employing reverse MR to explore their potential mediation effects. To investigate the role of metabolites as mediators, a two-step MR strategy was utilized (27). Multiple sensitivity analyses were conducted to verify the robustness of our findings, as depicted in the study’s design and progression in **Figure 1**.

### 2.2 Data sources of exposures, mediators, and outcomes

The data on exposures, mediators, and outcomes were obtained from GWAS, predominantly involving individuals of European ancestry. Immune cell data were derived from the dataset by Orrù V et al. (28), encompassing 731 immune cell traits from 3757 participants. The GWAS Catalog provided statistical summaries (accession numbers: GCST90001391-90002121, **Supplementary Table 1**). Metabolite data, including 1,091 metabolites and 309 metabolite ratios, organized into eight recognized pathways such as lipids, amino acids, and more, were also sourced from the GWAS Catalog (accession numbers: GCST90199621-90201020, **Supplementary Table 2**) (28). CRC data originated from a meta-analysis by Sakaue S et al. (29), which included 470,002 European participants, comprising 6,581 cases and 463,421 controls (GWAS Catalog accession number: GCST90018808).

### 2.3 SNP selection

**Figure 2:**
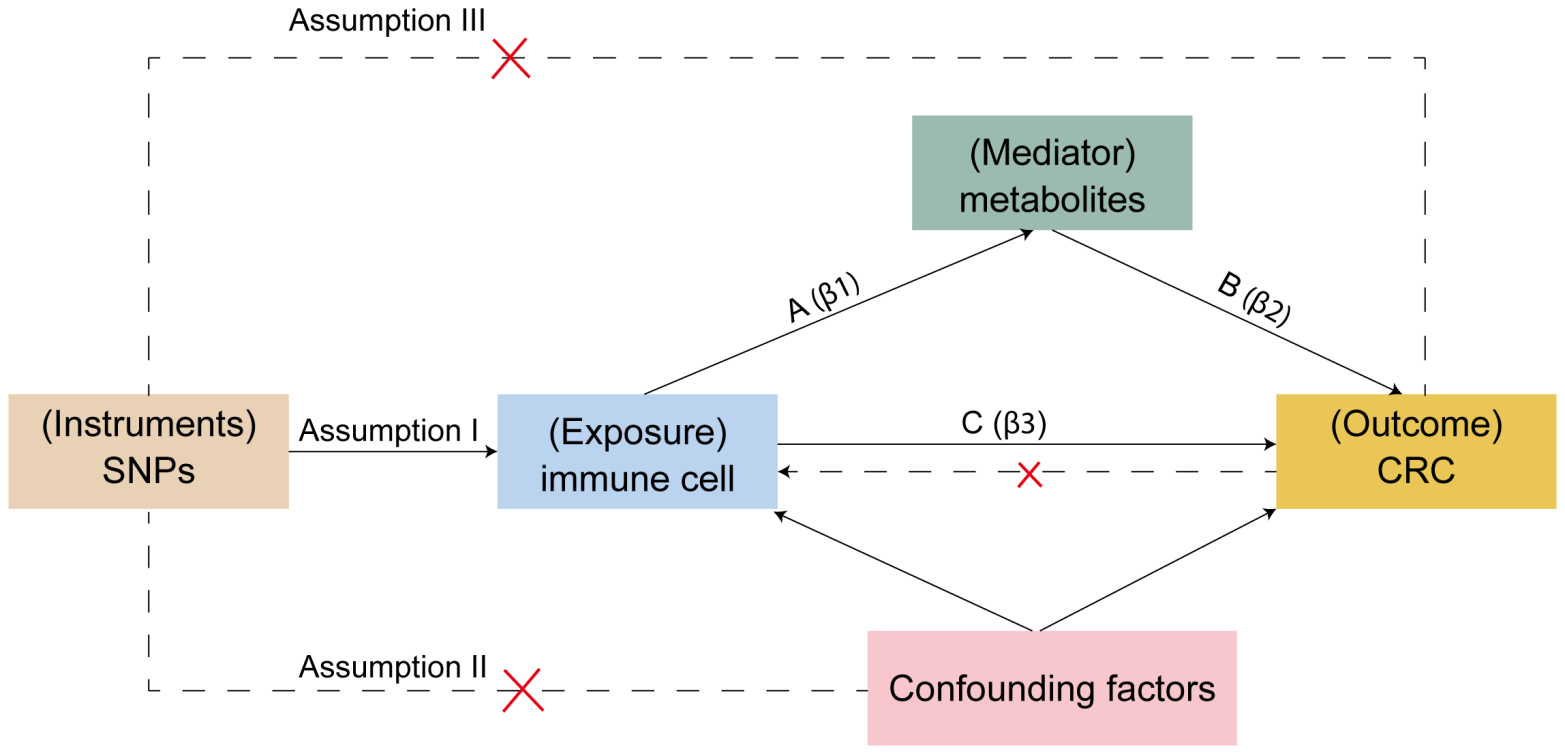
Mendelian randomization assumptions.

For Mendelian randomization studies to be valid, the single nucleotide polymorphisms (SNPs) serving as instrumental variables (IVs) must meet three crucial criteria (**Figure 2**): (1) SNPs must be correlate with the exposure, (2) SNPs should have no association with confounding elements like age, sex, or lifestyle, and (3) SNPs must exclusively affect the outcome via the exposure. The first assumption can be tested by selecting genetic variants significantly associated with risk factors in GWAS. The validity of the second assumption can be assessed by examining whether genetic variants are associated with competing risk factors. The third assumption cannot be directly assessed but must be supported by biological knowledge (30). SNPs were chosen based on their association with immune cell traits at a genome-wide significance threshold of *P* < 1 × 10^-5^ (31, 32), the criteria used for selecting significant SNPs in the reverse Mendelian randomization analysis were *P* < 5 × 10^-8^; independence was verified by assessing linkage disequilibrium, selecting IVs with no linkage effects (r^2^ < 0.001 within a 10,000 kb distance) (33). IVs were then screened from the CRC dataset to exclude any associated directly with the outcome and to remove palindromic SNPs. The strength of individual SNPs was determined by calculating the F-statistic, retaining those with an F-statistic greater than 10 (34). SNPs for immune cells and metabolites are listed in **Supplementary Tables 3, 4**.

### 2.4 Mendelian randomization and statistical analysis

A bidirectional two-sample MR analysis was performed using R (version 4.3.1) and the “TwoSampleMR” package to evaluate the link between immune cells and CRC. The impact on CRC risk was quantified as odds ratios (OR) with 95% confidence intervals (CI), considering a *P*-value < 0.05 as indicative of a significant causal connection. The main method employed was a random-effects inverse variance weighted (IVW) analysis, enhanced with MR-Egger, weighted median, simple mode, and weighted mode approaches to ensure result consistency.

### 2.5 Sensitivity analysis

The primary assessment of the impact of immune cells on CRC employed the random-effects IVW method (35). Up to four MR methods, each with different assumptions regarding pleiotropy, were applied to generate effect estimates. Significant heterogeneity was identified through Cochran’s Q test, with a *P*-value below 0.05 indicating its significance. Pleiotropy was assessed using the MR-PRESSO test and MR-Egger regression intercept, with *P*-values above 0.05 suggesting no pleiotropy. The robustness of our findings was further confirmed by a leave-one-out analysis, which assessed the impact of individual SNPs on the MR analysis.

### 2.6 Mediation analysis

A two-step MR approach was executed to investigate if an intermediate risk factor mediates the link between immune cells and CRC. First, we used bidirectional Mendelian randomization to identify 23 immune cells with a causal relationship to CRC out of 731 immune cells. The criteria for SNP selection included a significance threshold of P < 1 × 10^-5^, exclusion of SNPs in linkage disequilibrium (r^2^ < 0.001 within a 10,000 kb distance), and removal of weak instruments (F < 10). Next, we applied the same criteria to identify 17 metabolites out of 1,400 with a causal relationship to CRC. We then used the 23 immune cell traits as exposure factors to identify significant mediators among the 17 metabolites, using the same SNP selection criteria. We employed the Inverse-Variance Weighted (IVW) method as the primary approach to estimate the effect of immune cells on metabolites (β1). The most significant metabolites associated with immune cell traits were then analyzed as mediators in the causal relationship with CRC. Ultimately, we identified the role of nine immune cell types in colorectal cancer risk, along with the mediating effects of nine metabolites. The direct effects β1 from immune cell traits to metabolites, β2 from metabolites to CRC, and the total effect β3 from immune cell traits to CRC were calculated. The mediating effect was defined as β = β1 * β2, and its proportion relative to the total effect was expressed as R = (β/β3) * 100% (36, 37).

## 3 Results

### 3.1 Two-sample Mendelian randomization analysis between immune cells and CRC

**Figure 3:**
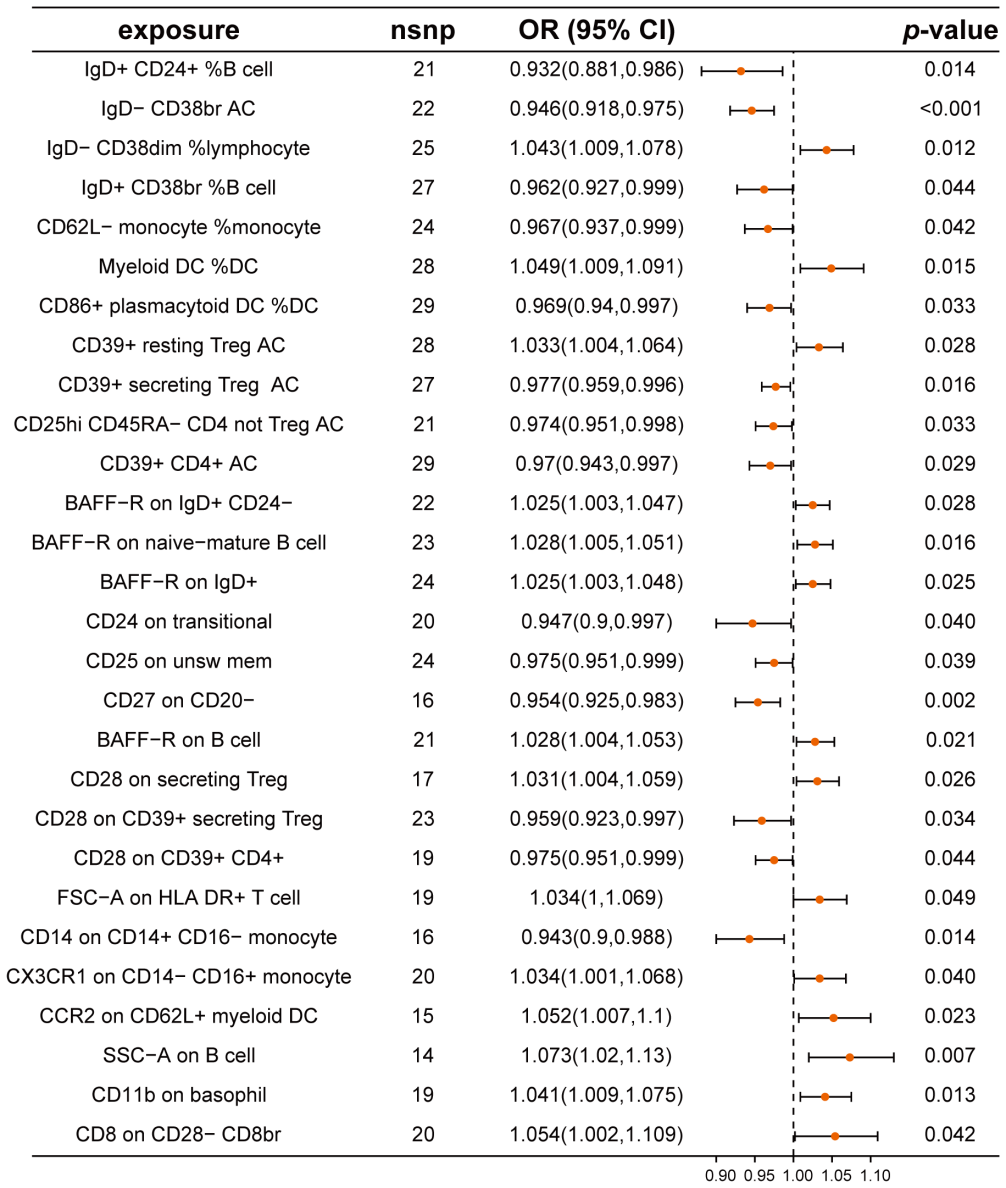
Mendelian randomization analysis between immune cells and CRC. This plot visualizes the association between immune cell and CRC. Each point denotes the Odds Ratio (OR) for the exposure. Horizontal lines represent the 95% confidence intervals. The vertical dashed line at OR=1 serves as a reference for no effect. OR > 1 indicates that immune cells are a risk factor for CRC, while OR < 1 indicates that immune cells are a protective factor for CRC.

We assessed the causal relationships between 28 types of immune cells and CRC using a two-sample MR approach (**Figure 3**; **Supplementary Table 5**). Our analysis distinguished 14 immune cell types as protective and 14 as risk factors for CRC. Notably, immune cells such as IgD^+^ CD24^+^ %B cell, CD14 on CD14^+^ CD16^-^ monocytes, and IgD^-^ CD38br AC exhibited odds ratios (ORs) of 0.932 (95% CI: 0.881-0.986), 0.943 (95% CI: 0.900-0.988), and 0.946 (95% CI: 0.918-0.975), respectively, indicating their protective roles against CRC. Conversely, SSC-A on B cells, CD8 on CD28^-^ CD8br, and CCR2 on CD62L^+^ myeloid DC were identified as risk factors, with ORs of 1.073 (95% CI: 1.020-1.130), 1.054 (95% CI: 1.002-1.109), and 1.052 (95% CI: 1.007-1.100), respectively. Our analyses for heterogeneity and pleiotropy showed no significant findings, confirming the robustness of our results (**Supplementary Table 6**).

Further validation through reverse Mendelian randomization analysis on these 28 immune cell types revealed no significant findings for 23 of these types (**Supplementary Table 7**), which were subsequently analyzed for potential mediating effects.

### 3.2 Effect of metabolites on CRC

**Figure 4:**
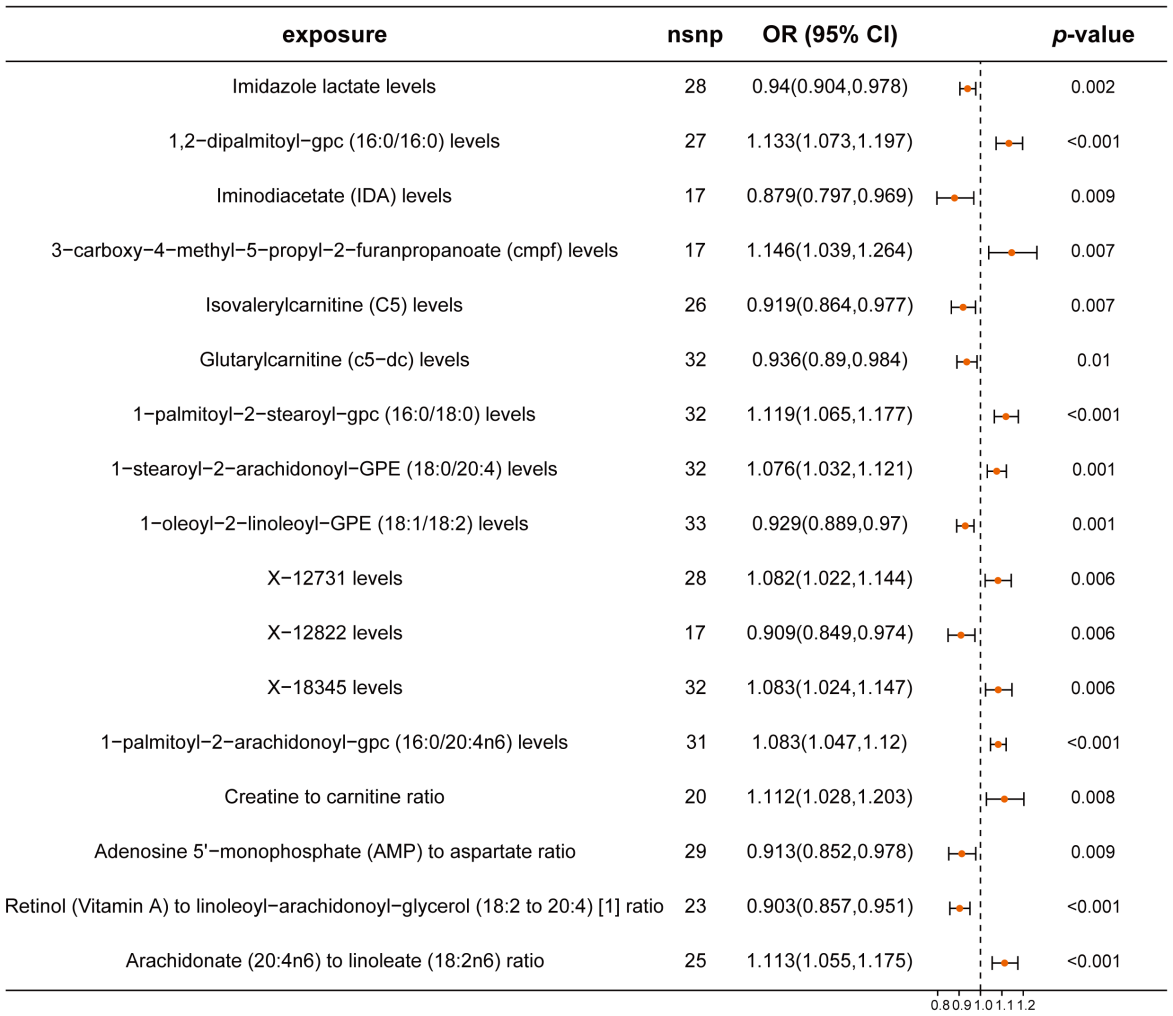
Forest plot of metabolites associated with CRC risk. This plot visualizes the association between metabolites and CRC. Each point denotes the Odds Ratio (OR) for the exposure. Horizontal lines represent the 95% confidence intervals. The vertical dashed line at OR=1 serves as a reference for no effect. OR > 1 indicates that metabolites are a risk factor for CRC, while OR < 1 indicates that metabolites are a protective factor for CRC.

In our mediation analysis, we explored the effects of 1,400 metabolites on CRC using the IVW method. We identified eight metabolites that demonstrated protective effects against CRC. Notably, IDA, an amino acid derivative, had a significant OR of 0.879 (95% CI: 0.797-0.969). Additionally, the ratio of retinol (Vitamin A) to linoleoyl-arachidonoyl-glycerol (18:2 to 20:4) showed a protective effect with an OR of 0.903 (95% CI: 0.857-0.951). Conversely, nine metabolites were identified as risk factors for CRC, including 3-carboxy-4-methyl-5-propyl-2-furanpropanoate (CMPF) and 1,2-dipalmitoyl-gpc (16:0/16:0), with ORs of 1.146 (95% CI: 1.039-1.264) and 1.133 (95% CI: 1.073-1.197), respectively (**Figure 4**; **Supplementary Table 8**). These findings highlight the complex relationship between specific metabolites and CRC and provide a basis for further mediation analysis. Analyses for heterogeneity and pleiotropy also indicated robustness (**Supplementary Table 9**).

### 3.3 Effect of immune cell traits on metabolites

**Figure 5:**
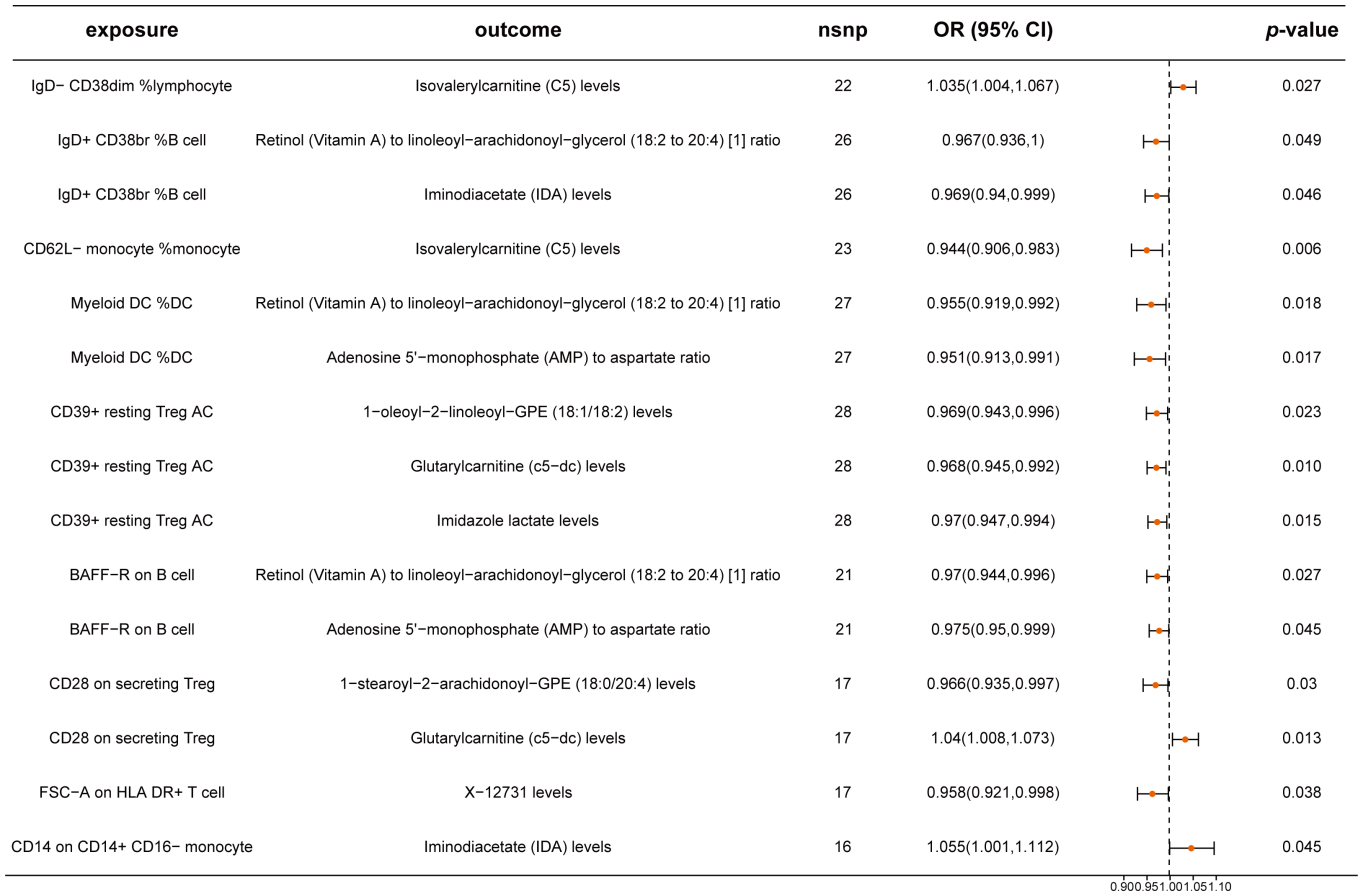
Mendelian randomization analysis between immune cells and metabolites. This plot visualizes the association between immune cells and metabolites. Each point denotes the Odds Ratio (OR) for the exposure. Horizontal lines represent the 95% confidence intervals. The vertical dashed line at OR=1 serves as a reference for no effect. OR > 1 indicates that immune cells are a risk factor for metabolites, while OR < 1 indicates that immune cells are a protective factor for metabolites.

Building on the previous identification of relationships between 23 immune cell traits and CRC, we examined the causal influence of these immune cell traits on 17 metabolites. Our analysis identified significant causal relationships for nine immune cells affecting nine metabolites (**Figure 5**; **Supplementary Table 10**). Notably, traits such as IgD^-^ CD38dim %lymphocyte for Isovalerylcarnitine (C5), CD28 on secreting Treg for Glutarylcarnitine (c5-dc), and CD14 on CD14^+^ CD16^-^ monocytes for Iminodiacetate (IDA) had ORs of 1.035 (95% CI: 1.004-1.067), 1.040 (95% CI: 1.008-1.073), and 1.055 (95% CI: 1.001-1.112), respectively. The remaining immune cell traits exhibited protective effects on specific metabolites. Our analyses confirmed no significant pleiotropy or heterogeneity, reinforcing the reliability of these results (**Supplementary Table 11**).

### 3.4 Mediation analysis of immune cell traits, metabolites, and colorectal cancer

**Figure 6:**
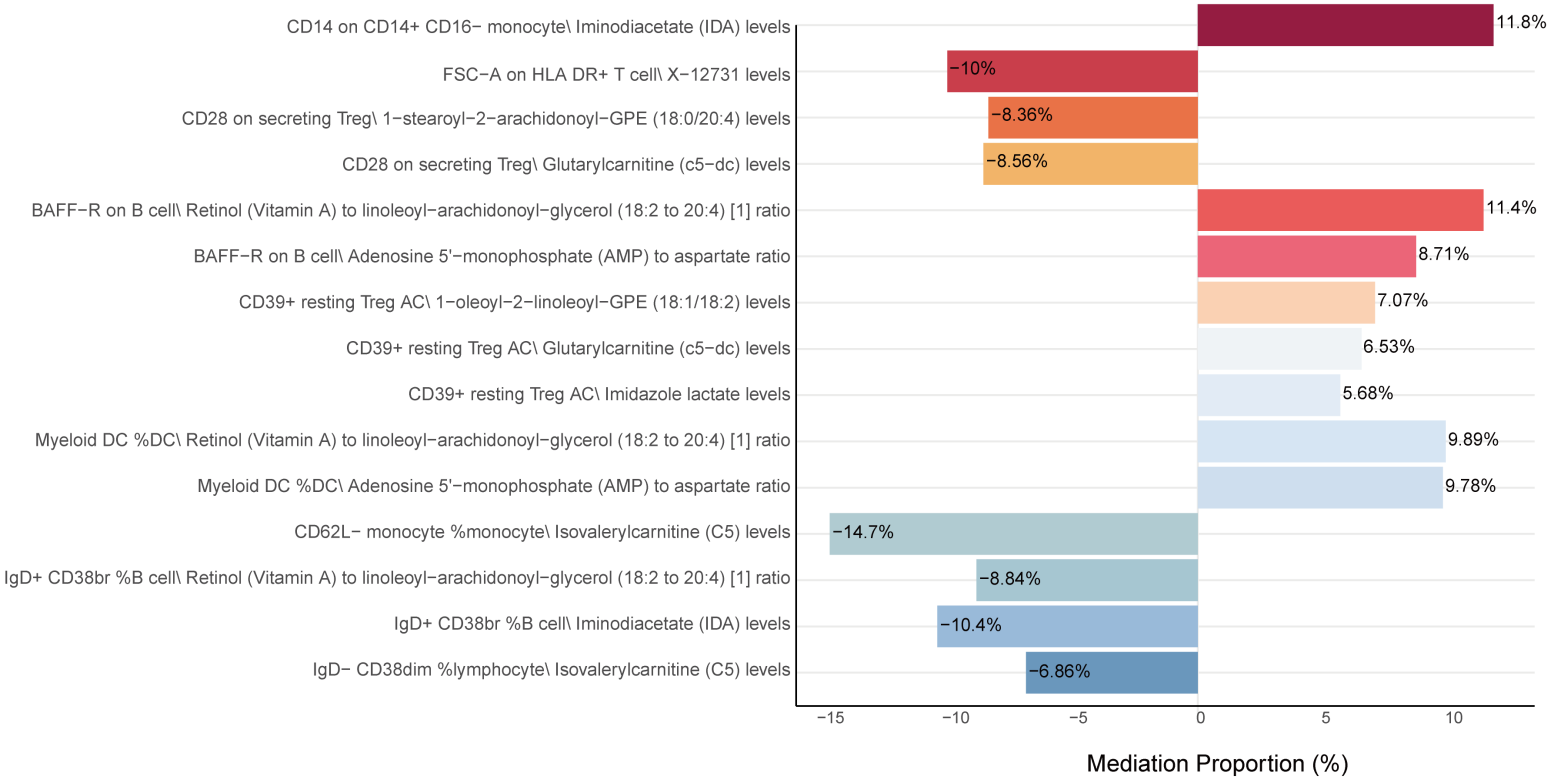
Mediation analysis of metabolites between immune cells and CRC. This figure shows the proportion of the mediation effect of each metabolite on the impact of various immune cells on CRC. A positive value indicates that the metabolite and immune cells have a consistent effect on CRC, while a negative value indicates that the metabolite and immune cells have opposite effects on CRC. The length of the bar represents the magnitude of the mediation effect.

After identifying key mediators affecting CRC and the effects of immune cell traits on these mediators, we quantified the proportion of mediating effects. Comprehensive results can be found in **Figure 6**, **Supplementary Table 12**, leave-one-out plot was presented in **Supplementary Figure 1**. Notably, IgD^+^ CD38br %B cell and CD14 on CD14^+^ CD16^-^ monocyte mediated their effects on CRC through IDA levels, with mediation proportions of -10.4% and 11.8%, respectively. Additionally, IgD^-^ CD38dim% lymphocyte and CD62L^-^ monocyte %monocyte mediated their effects through isovalerylcarnitine (C5) levels, with mediation proportions of -6.86% and -14.7%, respectively. Furthermore, IgD^+^ CD38br %B cell and BAFF-R on B cell mediated their effects through the Retinol (Vitamin A) to linoleoyl-arachidonoyl-glycerol (18:2 to 20:4) ratio, with mediation proportions of -8.84% and 11.4%, respectively.

## 4 Discussion

In this MR study examining the association between immune cells and CRC risk, we identified 23 immune cell types linked to CRC, with specific blood metabolites potentially acting as mediators. For example, The ratios of Adenosine 5’-monophosphate (AMP) to aspartate and Retinol (Vitamin A) to linoleoyl-arachidonoyl-glycerol (18:2 to 20:4) were found to concurrently mediate the promoting effects of Myeloid DC %DC and BAFF-R on B cells in colorectal cancer (CRC). IDA mediates the protective effect of CD14^+^ CD16^-^ monocytes on CRC risk with a mediation effect proportion of 11.8%. Conversely, IDA was also seen to decrease the protective effect of IgD^+^ CD38br %B cells on CRC risk, with a mediation effect proportion of -10.4%. Additionally, CD39^+^ resting Treg cells increase CRC risk through metabolites such as imidazole lactate, glutarylcarnitine (C5-DC), and 1-oleoyl-2-linoleoyl-GPE (18:1/18:2), with mediation proportions of 5.68%, 6.53%, and 7.07%, respectively. These results underscore the intricate interactions between specific immune cells, their associated metabolites, and their cumulative impact on CRC, highlighting the importance of understanding both direct and indirect effects of immune cell traits on CRC to develop targeted therapeutic strategies.

Immune cells hold significant prognostic value in colorectal cancer (CRC), with their plasticity within the tumor microenvironment (TME) leading to the expression of various phenotypes that either promote or inhibit tumor progression. Our study identifies four dendritic cell (DC) subsets associated with CRC development: Myeloid DC %DC and CCR2 on CD62L^+^ myeloid DC are risk factors for CRC, while CD86^+^ plasmacytoid DC %DC and CD62L^-^ monocyte %monocyte serve as protective factors. This aligns with findings by Gulubova MV, who reported that higher infiltration of CD83^+^ DCs correlates with better prognosis in CRC patients (38). Conversely, Sandel MH observed that CD208-positive and CD1a-positive infiltrating dendritic cells are associated with shortened survival in CRC patients (39). Further mediation analysis in our study reveals that the ratios of Adenosine 5’-monophosphate (AMP) to aspartate and Retinol (Vitamin A) to linoleoyl-arachidonoyl-glycerol (18:2 to 20:4) mediate the promoting effects of Myeloid DC %DC on CRC. Additionally, Isovalerylcarnitine (C5) levels diminish the protective role of CD62L^-^ monocyte %monocyte against CRC, suggesting that retinol metabolism, lipid metabolism, and leucine metabolism in DC cells impact CRC progression. Previous studies have highlighted the critical role of metabolic regulation in DC function. Alterations in cholesterol metabolism and mevalonate (MVA) signaling can affect DC function and consequently the immune response of CRC (40). The accumulation of lipid droplets can impede calreticulin exposure, thus preventing DC maturation, delaying DC activation, and promoting CRC progression (41). However, reports on the impact of vitamin A and leucine metabolism in DC cells on CRC are lacking.

Our study also reveals differential impacts of B cell phenotypes on CRC. IgD^+^ CD24^+^ %B cells, IgD^-^ CD38br AC, and IgD^+^ CD38br %B cells act as protective factors against CRC, while IgD^-^ CD38dim %lymphocyte, BAFF-R on IgD^+^ CD24^-^, BAFF-R on naive-mature B cell, BAFF-R on IgD^+^, BAFF-R on B cell, and SSC-A on B cell are identified as risk factors. Mediation analysis further shows that Iminodiacetate (IDA) levels and Retinol (Vitamin A) to linoleoyl-arachidonoyl-glycerol (18:2 to 20:4) ratio attenuate the protective effect of IgD^+^ CD38br %B cells on CRC, whereas Isovalerylcarnitine (C5) levels diminish the promoting effect of IgD^-^ CD38dim %lymphocyte. Besides, the ratios of Adenosine 5’-monophosphate (AMP) to aspartate and Retinol (Vitamin A) to linoleoyl-arachidonoyl-glycerol (18:2 to 20:4) mediate the promoting effects of BAFF-R on B cells in CRC. Our findings suggest that most B cells contribute to immune escape in CRC, consistent with previous reports indicating that certain regulatory B cell subsets, which preferentially metabolize leucine, can promote CRC progression (42). Lipid metabolites such as acylcarnitine enhance B cell-mediated anti-tumor immunity by promoting mitochondrial oxidative phosphorylation (43). Fundamental research indicates that inhibiting the conversion of B cells to IgA^+^ cells may suppress CRC growth (44). In contrast, previous Mendelian randomization studies showed that IgD^+^CD24^+^ B cells are risk factors for glioblastoma (45), suggesting differences across tumor types and necessitating further experimental research to elucidate the roles of various immune cell phenotypes in CRC development and progression.

Moreover, we identified CD39^+^ resting Treg cells and CD28 on secreting Treg cells as risk factors for CRC, consistent with previous findings that increased accumulation of Treg cells is often associated with CRC progression, metastasis, immune therapy failure, and poor prognosis (46–49). Additionally, our study found that Imidazole lactate levels, Glutarylcarnitine (C5-DC) levels, and 1-oleoyl-2-linoleoyl-GPE (18:1/18:2) levels mediate the promoting effect of CD39^+^ resting Treg cells on CRC, while Glutarylcarnitine (C5-DC) levels and 1-stearoyl-2-arachidonoyl-GPE (18:0/20:4) levels attenuate the promoting effect of CD28 on secreting Treg cells. This suggests that amino acid metabolism and fatty acid oxidation may play roles in regulating Treg cells’ impact on CRC. Previous research has shown that kynurenine can enhance tumor aggressiveness by upregulating PD-L1 expression on Treg cells, thus contributing to immune escape in CRC (50). Additionally, TI-Treg cells use lipid metabolism-driven oxidative phosphorylation (OXPHOS) to meet their energy needs and immune suppressive functions (51). Furthermore, high IDO expression in CRC cells and tryptophan depletion in the TME are associated with tumor immune escape and increased Treg infiltration in CRC (52). The above findings suggest that targeting amino acid and fatty acid metabolites of different Treg cell subtypes could offer a potential strategy for immune regulation in CRC.

Substantial evidence suggests that blood metabolites are closely linked to tumor malignancy. For instance, ketogenic diets rich in β-hydroxybutyrate can inhibit CRC cell growth (53), and α-ketoglutarate in the TME suppresses tumor growth (54). Beyond direct effects on tumor cells, metabolites like indole-3-propionic acid (IPA) regulate CD8^+^ T cell stemness and enhance anti-PD-1 immunotherapy efficacy (55). Lactate in the TME also boosts CD8^+^ T cell stemness and anti-tumor immunity (56), while gut microbiota-derived butyrate enhances chemotherapy efficacy by modulating CD8^+^ T cell function (57), and indole-3-carboxylic acid (ICA) augments CD8^+^ T cell functionality by inhibiting regulatory T cell differentiation (58). We identified 17 metabolites associated with CRC risk, with eight reducing and nine increasing CRC risk. Yet, there is no experimental evidence directly linking IDA, CMPF, or 1,2-dipalmitoyl-glycerophosphocholine to tumorigenesis.

This study leverages large-scale GWAS data to conduct two-sample, two-step, and mediation MR analyses, emphasizing the methodological rigor and efficiency of our approach. Our conclusions, founded on genetic instrumental variables and supported by extensive pleiotropy analyses, are reliable and minimally affected by horizontal pleiotropy or other confounding factors. However, the study has limitations, First, The MR-Egger and weighted median MR approaches might have lacked sufficient power to identify directional pleiotropy in the genetic instruments, if it existed (59). Second, Although MR can mitigate confounding effects from environmental and behavioral factors, residual confounding remains unavoidable. Third, notably the focus on European populations, which may limit the generalizability of the findings. Fourth, our reliance on summary-level data precludes detailed causal relationship analyses within specific subgroups, such as gender or cancer subtype. Despite these limitations, our comprehensive insights provide a solid foundation for understanding the complex dynamics at play and the potential for developing targeted therapeutic strategies based on these findings.

## 5 Conclusion

In conclusion, this study provides a comprehensive evaluation of the roles of circulating immune cells and blood metabolites in the development of CRC, uncovering significant abnormalities in the immune-metabolic network associated with this disease. Our findings open new avenues for understanding the pathogenesis of CRC and highlight the critical role of blood metabolites in mediating the relationship between immune cells and CRC. This enhanced understanding of CRC’s pathological processes not only deepens our knowledge of the disease but also lays a theoretical foundation for the development of novel immunotherapeutic and metabolite-targeted interventions. These insights are pivotal for advancing CRC treatment strategies, emphasizing the necessity of further research to explore these complex biological interactions and their potential therapeutic applications.

## Data Availability

The original contributions presented in the study are included in the article/**Supplementary Material**. Further inquiries can be directed to the corresponding authors.

## Supplementary material

Supplementary Figure 1 Leave-one-out plots for the mediation analysis.

## References

[1] Bray F Laversanne M Sung H Ferlay J Siegel RL Soerjomataram I. Global cancer statistics 2022: GLOBOCAN estimates of incidence and mortality worldwide for 36 cancers in 185 countries. CA Cancer J Clin. (2024) 74:229–63. doi: 10.3322/caac.21834 38572751

[2] Siegel RL Miller KD Wagle NS Jemal A. Cancer statistics, 2023. CA Cancer J Clin. (2023) 73:17–48. doi: 10.3322/caac.21763 36633525

[3] Chung DC Rustgi AK. The hereditary nonpolyposis colorectal cancer syndrome: genetics and clinical implications. Ann Intern Med. (2003) 138:560–70. doi: 10.7326/0003-4819-138-7-200304010-00012 12667026

[4] Jess T Rungoe C Peyrin-Biroulet L. Risk of colorectal cancer in patients with ulcerative colitis: a meta-analysis of population-based cohort studies. Clin Gastroenterol Hepatol. (2012) 10:639–45. doi: 10.1016/j.cgh.2012.01.010 22289873

[5] Botteri E Borroni E Sloan EK Bagnardi V Bosetti C Peveri G. Smoking and colorectal cancer risk, overall and by molecular subtypes: A meta-analysis. Am J Gastroenterol. (2020) 115:1940–9. doi: 10.14309/ajg.0000000000000803 32773458

[6] Fedirko V Tramacere I Bagnardi V Rota M Scotti L Islami F. Alcohol drinking and colorectal cancer risk: an overall and dose-response meta-analysis of published studies. Ann Oncol. (2011) 22:1958–72. doi: 10.1093/annonc/mdq653 21307158

[7] Kyrgiou M Kalliala I Markozannes G Gunter MJ Paraskevaidis E Gabra H. Adiposity and cancer at major anatomical sites: umbrella review of the literature. BMJ. (2017) 356:j477. doi: 10.1136/bmj.j477 28246088 PMC5421437

[8] Wong CC Yu J. Gut microbiota in colorectal cancer development and therapy. Nat Rev Clin Oncol. (2023) 20:429–52. doi: 10.1038/s41571-023-00766-x 37169888

[9] Fang M Li Y Huang K Qi S Zhang J Zgodzinski W. IL33 promotes colon cancer cell stemness via JNK activation and macrophage recruitment. Cancer Res. (2017) 77:2735–45. doi: 10.1158/0008-5472.CAN-16-1602 PMC5760170 28249897

[10] Kießler M Plesca I Sommer U Wehner R Wilczkowski F Müller L. Tumor-infiltrating plasmacytoid dendritic cells are associated with survival in human colon cancer. J Immunother Cancer. (2021) 9:e001813. doi: 10.1136/jitc-2020-001813 33762320 PMC7993360

[11] Sundström P Stenstad H Langenes V Ahlmanner F Theander L Ndah TG. Regulatory T cells from colon cancer patients inhibit effector T-cell migration through an adenosine-dependent mechanism. Cancer Immunol Res. (2016) 4:183–93. doi: 10.1158/2326-6066.CIR-15-0050 26787824

[12] Toffoli EC van Vliet AA Verheul HWM van der Vliet HJ Tuynman J Spanholtz J. Allogeneic NK cells induce monocyte-to-dendritic cell conversion, control tumor growth, and trigger a pro-inflammatory shift in patient-derived cultures of primary and metastatic colorectal cancer. J Immunother Cancer. (2023) 11:e007554. doi: 10.1136/jitc-2023-007554 38056896 PMC10711876

[13] Leone RD Powell JD. Metabolism of immune cells in cancer. Nat Rev Cancer. (2020) 20:516–31. doi: 10.1038/s41568-020-0273-y PMC8041116 32632251

[14] Sukumar M Liu J Ji Y Subramanian M Crompton JG Yu Z. Inhibiting glycolytic metabolism enhances CD8^+^ T cell memory and antitumor function. J Clin Invest. (2013) 123:4479–88. doi: 10.1172/JCI69589 PMC3784544 24091329

[15] Geiger R Rieckmann JC Wolf T Basso C Feng Y Fuhrer T. L-arginine modulates T cell metabolism and enhances survival and anti-tumor activity. Cell. (2016) 167:829–42.e13. doi: 10.1016/j.cell.2016.09.031 PMC5075284 27745970

[16] Nabe S Yamada T Suzuki J Toriyama K Yasuoka T Kuwahara M. Reinforce the antitumor activity of CD8^+^ T cells via glutamine restriction. Cancer Sci. (2018) 109:3737–50. doi: 10.1111/cas.13827 PMC6272119 30302856

[17] Yang W Bai Y Xiong Y Zhang J Chen S Zheng X. Potentiating the antitumour response of CD8(^+^) T cells by modulating cholesterol metabolism. Nature. (2016) 531:651–5. doi: 10.1038/nature17412 PMC4851431 26982734

[18] Cong J Wang X Zheng X Wang D Fu B Sun R. Dysfunction of natural killer cells by FBP1-induced inhibition of glycolysis during lung cancer progression. Cell Metab. (2018) 28:243–55.e5. doi: 10.1016/j.cmet.2018.06.021 30033198

[19] Hossain F Al-Khami AA Wyczechowska D Hernandez C Zheng L Reiss K. Inhibition of fatty acid oxidation modulates immunosuppressive functions of myeloid-derived suppressor cells and enhances cancer therapies. Cancer Immunol Res. (2015) 3:1236–47. doi: 10.1158/2326-6066.CIR-15-0036 PMC4636942 26025381

[20] Zhu C-X Yan K Chen L Huang RR Bian ZH Wei HR. Targeting OXCT1-mediated ketone metabolism reprograms macrophages to promote antitumor immunity via CD8^+^ T cells in hepatocellular carcinoma. J Hepatol. (2024) S0168-8278(24)00342-8. doi: 10.1016/j.jhep.2024.05.007 38759889

[21] Goossens P Rodriguez-Vita J Etzerodt A Masse M Rastoin O Gouirand V. Membrane cholesterol efflux drives tumor-associated macrophage reprogramming and tumor progression. Cell Metab. (2019) 29:1376–89.e4. doi: 10.1016/j.cmet.2019.02.016 30930171

[22] Sekula P Del Greco MF Pattaro C Köttgen A. Mendelian randomization as an approach to assess causality using observational data. J Am Soc Nephrol. (2016) 27:3253–65. doi: 10.1681/ASN.2016010098 PMC5084898 27486138

[23] Ming R Wu H Liu H Zhan F Qiu X Ji M. Causal effects and metabolites mediators between immune cell and risk of breast cancer: a Mendelian randomization study. Front In Genet. (2024) 15:1380249. doi: 10.3389/fgene.2024.1380249 38826800 PMC11140059

[24] Zhang K Zhu J Wang P Chen Y Wang Z Ge X. Plasma metabolites as mediators in immune cell-pancreatic cancer risk: insights from Mendelian randomization. Front Immunol. (2024) 15:1402113. doi: 10.3389/fimmu.2024.1402113 38933268 PMC11199692

[25] Zhu X Qiu Z Yang M Yang J Kong L Li L. Causal associations of BAFF-R on IgD^+^ CD24- B cell immune cell trait with hepatocellular carcinoma and the mediating role of phenylacetylglutamate levels: a Mendelian randomization study. J Cancer. (2024) 15:4591–603. doi: 10.7150/jca.96059 PMC11242345 39006080

[26] Constantinescu A-E Bull CJ Jones N Mitchell R Burrows K Dimou N. Circulating white blood cell traits and colorectal cancer risk: A Mendelian randomisation study. Int J Cancer. (2024) 154:94–103. doi: 10.1002/ijc.34691 PMC10864681 37578112

[27] Carter AR Sanderson E Hammerton G Richmond RC Davey Smith G Heron J. Mendelian randomisation for mediation analysis: current methods and challenges for implementation. Eur J Epidemiol. (2021) 36:465–78. doi: 10.1007/s10654-021-00757-1 PMC8159796 33961203

[28] Orrù V Steri M Sidore C Marongiu M Serra V Olla S. Complex genetic signatures in immune cells underlie autoimmunity and inform therapy. Nat Genet. (2020) 52:1036–45. doi: 10.1038/s41588-020-0684-4 PMC8517961 32929287

[29] Sakaue S Kanai M Tanigawa Y Karjalainen J Kurki M Koshiba S. A cross-population atlas of genetic associations for 220 human phenotypes. Nat Genet. (2021) 53:1415–24. doi: 10.1038/s41588-021-00931-x PMC12208603 34594039

[30] Larsson SC Butterworth AS Burgess S. Mendelian randomization for cardiovascular diseases: principles and applications. Eur Heart J. (2023) 44:4913–24. doi: 10.1093/eurheartj/ehad736 PMC10719501 37935836

[31] Xiang Y Zhang C Wang J Cheng Y Wang L Tong Y. Identification of host gene-microbiome associations in colorectal cancer patients using mendelian randomization. J Transl Med. (2023) 21:535. doi: 10.1186/s12967-023-04335-9 37563724 PMC10416448

[32] Ni J-J Li X-S Zhang H Xu Q Wei XT Feng GJ. Mendelian randomization study of causal link from gut microbiota to colorectal cancer. BMC Cancer. (2022) 22:1371. doi: 10.1186/s12885-022-10483-w 36585646 PMC9804960

[33] Cao J Wang Z Zhu M Huang Y Jin Z Xiong Z. Low-density lipoprotein cholesterol and risk of hepatocellular carcinoma: a Mendelian randomization and mediation analysis. Lipids Health Dis. (2023) 22:110. doi: 10.1186/s12944-023-01877-1 37525197 PMC10388495

[34] Pierce BL Ahsan H Vanderweele TJ. Power and instrument strength requirements for Mendelian randomization studies using multiple genetic variants. Int J Epidemiol. (2011) 40:740–52. doi: 10.1093/ije/dyq151 PMC3147064 20813862

[35] Yun Z Guo Z Li X Shen Y Nan M Dong Q. Genetically predicted 486 blood metabolites in relation to risk of colorectal cancer: A Mendelian randomization study. Cancer Med. (2023) 12:13784–99. doi: 10.1002/cam4.6022 PMC10315807 37132247

[36] VanderWeele TJ. Mediation analysis: A practitioner’s guide. Annu Rev Public Health. (2016) 37:17–32. doi: 10.1146/annurev-publhealth-032315-021402 26653405

[37] Dai H Hou T Wang Q Hou Y Wang T Zheng J. Causal relationships between the gut microbiome, blood lipids, and heart failure: a Mendelian randomization analysis. Eur J Prev Cardiol. (2023) 30:1274–82. doi: 10.1093/eurjpc/zwad171 37195998

[38] Gulubova MV Ananiev JR Vlaykova TI Yovchev Y Tsoneva V Manolova IM. Role of dendritic cells in progression and clinical outcome of colon cancer. Int J Colorectal Dis. (2012) 27:159–69. doi: 10.1007/s00384-011-1334-1 22065108

[39] Sandel MH Dadabayev AR Menon AG Morreau H Melief CJ Offringa R. Prognostic value of tumor-infiltrating dendritic cells in colorectal cancer: role of maturation status and intratumoral localization. Clin Cancer Research: an Off J Am Assoc For Cancer Res. (2005) 11:2576–82. doi: 10.1158/1078-0432.CCR-04-1448 15814636

[40] Yang J Pan X Zhang J Ma S Zhou J Jia Z. Reprogramming dysfunctional dendritic cells by a versatile metabolism nano-intervenor for enhancing cancer combinatorial immunotherapy. Nano Today. (2022) 46:101618. doi: 10.1016/j.nantod.2022.101618

[41] Cotte AK Aires V Fredon M Limagne E Derangère V Thibaudin M. Lysophosphatidylcholine acyltransferase 2-mediated lipid droplet production supports colorectal cancer chemoresistance. Nat Commun. (2018) 9:322. doi: 10.1038/s41467-017-02732-5 29358673 PMC5778070

[42] Wang Z Lu Z Lin S Xia J Zhong Z Xie Z. Leucine-tRNA-synthase-2-expressing B cells contribute to colorectal cancer immunoevasion. Immunity. (2022) 55:1067–81.e8. doi: 10.1016/j.immuni.2022.04.017 35659337

[43] Zhang Z Xu X Zhang D Zhao S Wang C Zhang G. Targeting Erbin-mitochondria axis in platelets/megakaryocytes promotes B cell-mediated antitumor immunity. Cell Metab. (2024) 36:541–56.e9. doi: 10.1016/j.cmet.2023.12.020 38232736

[44] Zhong Z Zhang H Nan K Zhong J Wu Q Lu L. Fasting-mimicking diet drives antitumor immunity against colorectal cancer by reducing igA-producing cells. Cancer Res. (2023) 83:3529–43. doi: 10.1158/0008-5472.CAN-23-0323 PMC10618736 37602826

[45] Huang M Liu Y Peng J Cheng Y. Causal effects of immune cells in glioblastoma: a Bayesian Mendelian Randomization study. Front Neurol. (2024) 15:1375723. doi: 10.3389/fneur.2024.1375723 38742049 PMC11089213

[46] Chang L-Y Lin Y-C Mahalingam J Huang CT Chen TW Kang CW. Tumor-derived chemokine CCL5 enhances TGF-β-mediated killing of CD8(^+^) T cells in colon cancer by T-regulatory cells. Cancer Res. (2012) 72:1092–102. doi: 10.1158/0008-5472.CAN-11-2493 22282655

[47] Brudvik KW Henjum K Aandahl EM Bjørnbeth BA Taskén K. Regulatory T-cell-mediated inhibition of antitumor immune responses is associated with clinical outcome in patients with liver metastasis from colorectal cancer. Cancer Immunology Immunotherapy: CII. (2012) 61:1045–53. doi: 10.1007/s00262-011-1174-4 PMC11029571 22159472

[48] Katz SC Bamboat ZM Maker AV Shia J Pillarisetty VG Yopp AC. Regulatory T cell infiltration predicts outcome following resection of colorectal cancer liver metastases. Ann Surg Oncol. (2013) 20:946–55. doi: 10.1245/s10434-012-2668-9 PMC3740360 23010736

[49] Chaput N Louafi S Bardier A Charlotte F Vaillant JC Ménégaux F. Identification of CD8^+^CD25^+^Foxp3^+^ suppressive T cells in colorectal cancer tissue. Gut. (2009) 58:520–9. doi: 10.1136/gut.2008.158824 19022917

[50] Deng B Yang B Chen J Wang S Zhang W Guo Y. Gallic acid induces T-helper-1-like Treg cells and strengthens immune checkpoint blockade efficacy. J Immunother Cancer. (2022) 10:e004037. doi: 10.1136/jitc-2021-004037 35817479 PMC9274539

[51] Michalek RD Gerriets VA Jacobs SR Macintyre AN MacIver NJ Mason EF. Cutting edge: distinct glycolytic and lipid oxidative metabolic programs are essential for effector and regulatory CD4^+^ T cell subsets. J Immunol. (2011) 186:3299–303. doi: 10.4049/jimmunol.1003613 PMC3198034 21317389

[52] Brandacher G Perathoner A Ladurner R Schneeberger S Obrist P Winkler C. Prognostic value of indoleamine 2,3-dioxygenase expression in colorectal cancer: effect on tumor-infiltrating T cells. Clin Cancer Research: an Off J Am Assoc For Cancer Res. (2006) 12:1144–51. doi: 10.1158/1078-0432.CCR-05-1966 16489067

[53] Dmitrieva-Posocco O Wong AC Lundgren P Golos AM Descamps HC Dohnalová L. [amp]]beta;-Hydroxybutyrate suppresses colorectal cancer. Nature. (2022) 605:160–5. doi: 10.1038/s41586-022-04649-6 PMC9448510 35477756

[54] Tran TQ Hanse EA Habowski AN Li H Ishak Gabra MB Yang Y. α-Ketoglutarate attenuates Wnt signaling and drives differentiation in colorectal cancer. Nat Cancer. (2020) 1:345–58. doi: 10.1038/s43018-020-0035-5 PMC7442208 32832918

[55] Jia D Wang Q Qi Y Jiang Y He J Lin Y. Microbial metabolite enhances immunotherapy efficacy by modulating T cell stemness in pan-cancer. Cell. (2024) 187:1651–65.e21. doi: 10.1016/j.cell.2024.02.022 38490195

[56] Feng Q Liu Z Yu X Huang T Chen J Wang J. Lactate increases stemness of CD8 ^+^ T cells to augment anti-tumor immunity. Nat Commun. (2022) 13:4981. doi: 10.1038/s41467-022-32521-8 36068198 PMC9448806

[57] He Y Fu L Li Y Wang W Gong M Zhang J. Gut microbial metabolites facilitate anticancer therapy efficacy by modulating cytotoxic CD8^+^ T cell immunity. Cell Metab. (2021) 33:988–1000.e7. doi: 10.1016/j.cmet.2021.03.002 33761313

[58] Fong W Li Q Ji F Liang W Lau HCH Kang X. Lactobacillus gallinarum-derived metabolites boost anti-PD1 efficacy in colorectal cancer by inhibiting regulatory T cells through modulating IDO1/Kyn/AHR axis. Gut. (2023) 72:2272–85. doi: 10.1136/gutjnl-2023-329543 PMC10715476 37770127

[59] Bowden J Davey Smith G Burgess S. Mendelian randomization with invalid instruments: effect estimation and bias detection through Egger regression. Int J Epidemiol. (2015) 44:512–25. doi: 10.1093/ije/dyv080 PMC4469799 26050253

