# Supplementary figures and images for: Causal effects and metabolites mediators between immune cell and risk of colorectal cancer: a Mendelian randomization study
Source: Front Immunol. 2024 Sep 12;15:1444222. doi: 10.3389/fimmu.2024.1444222 (PMC11428109; doi:10.3389/fimmu.2024.1444222)

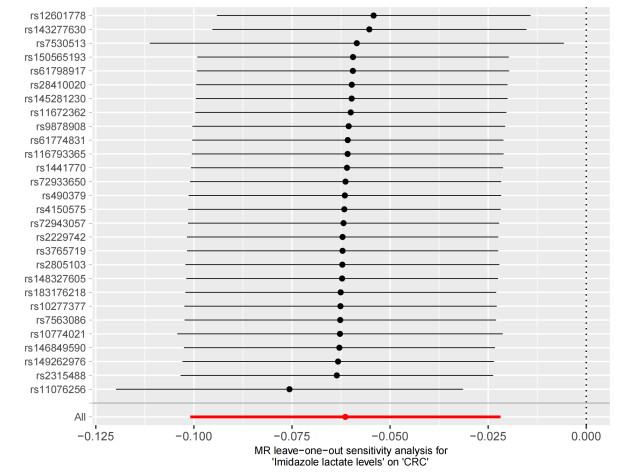

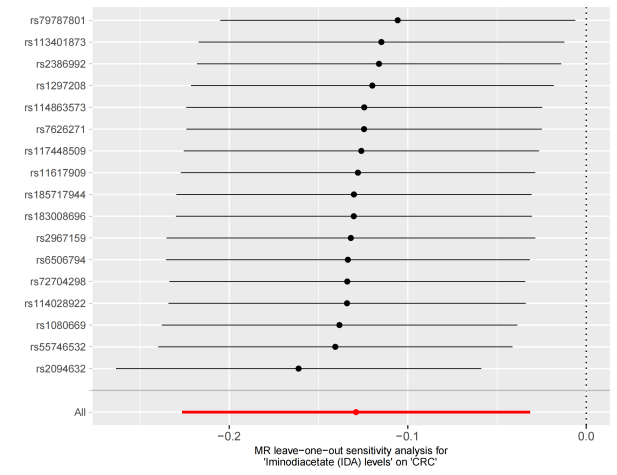

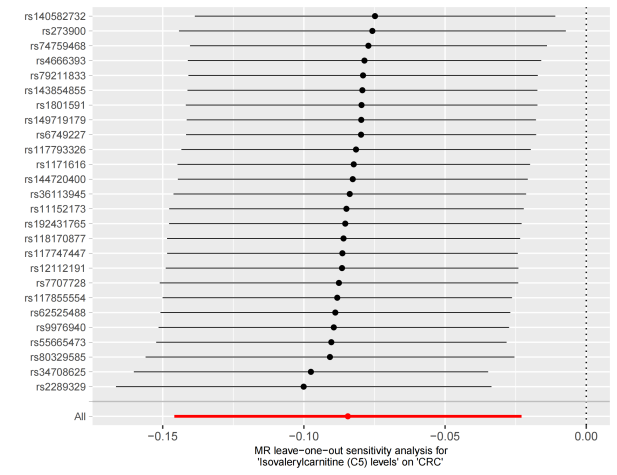

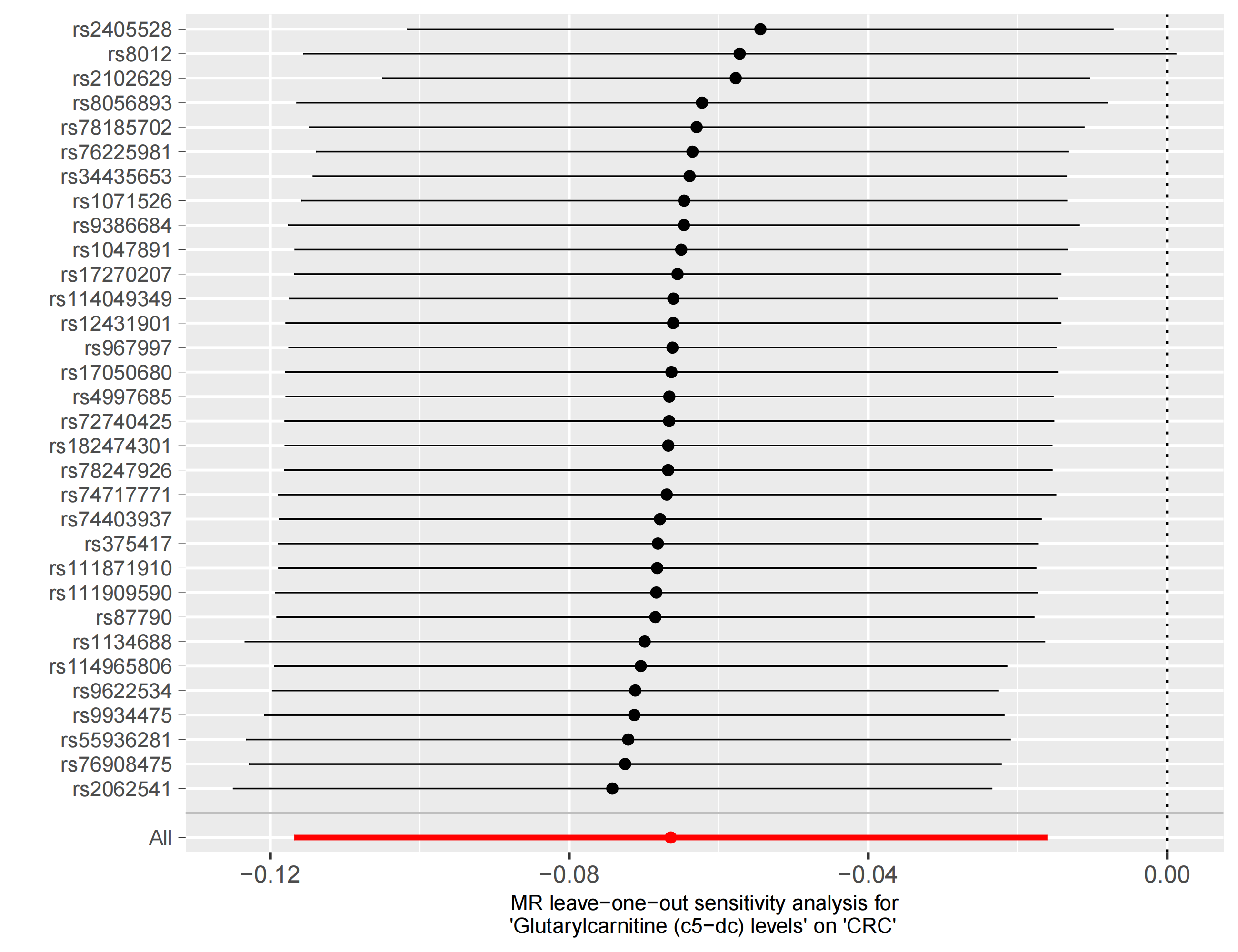

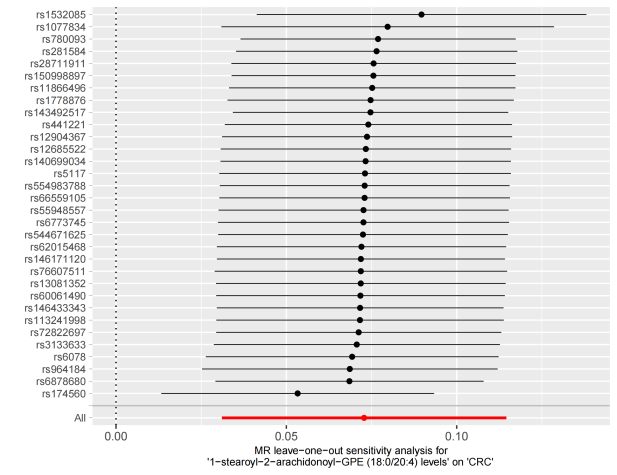

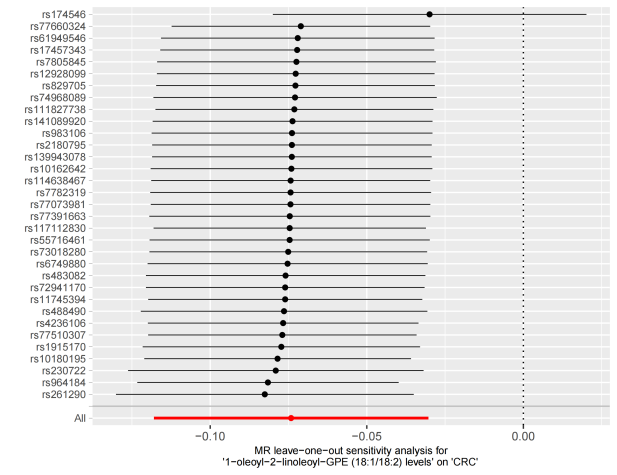

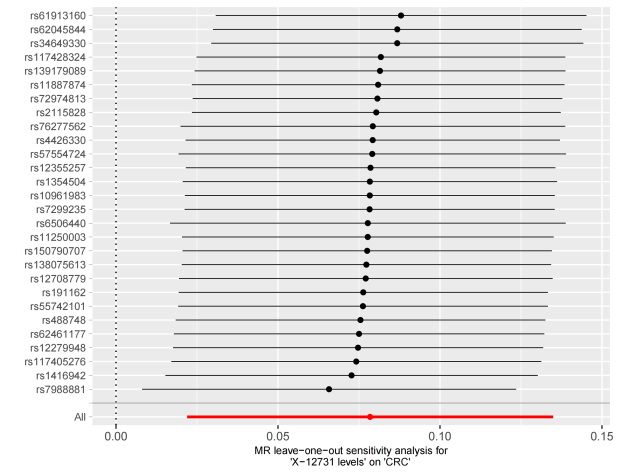

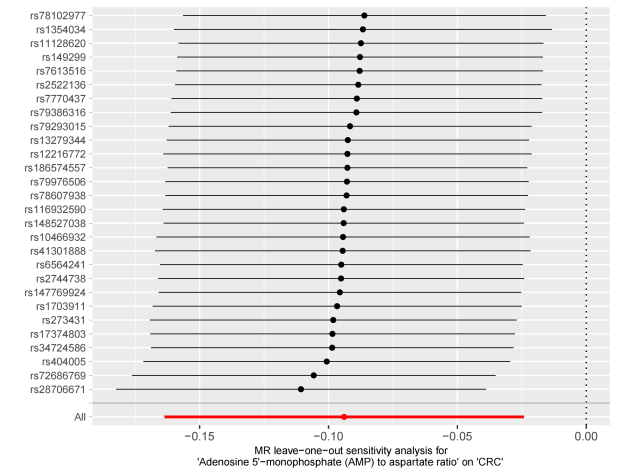

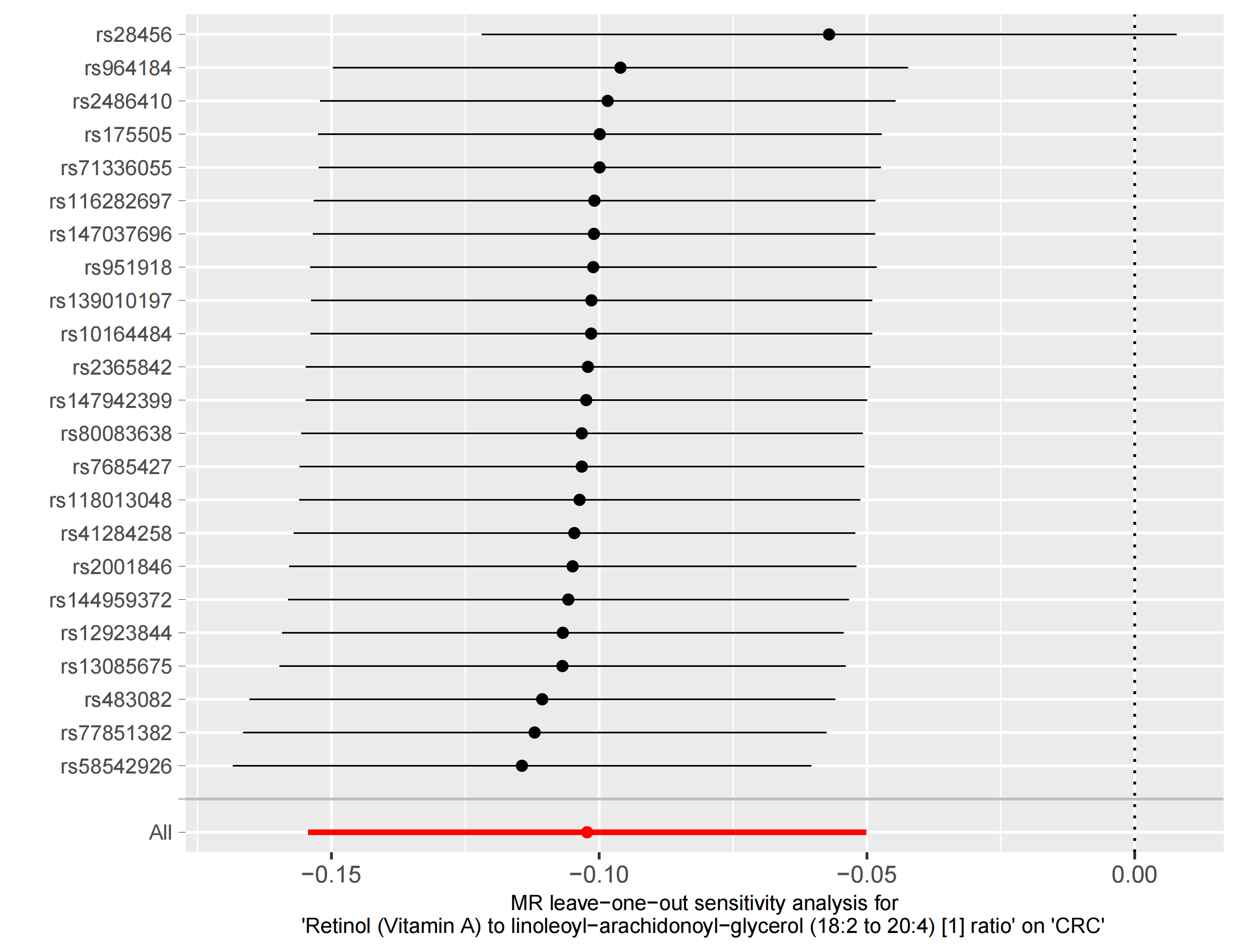

Supplement: Supplementary Figure 1 — Leave-one-out plots for the mediation analysis. [file DataSheet1.docx]
